# Can Anorectal Atresia Be Diagnosed in the First Trimester of Pregnancy? A Systematic Literature Review

**DOI:** 10.3390/medicina56110583

**Published:** 2020-10-30

**Authors:** Liana Ples, Radu Chicea, Mircea-Octavian Poenaru, Adrian Neacsu, Romina Marina Sima, Romeo Micu

**Affiliations:** 1 Department of Obstetrics and Gynecology, Carol Davila University of Medicine and Pharmacy, 050474 Bucharest, Romania; liana.ples@umfcd.ro (L.P.); mircea.poenaru@umfcd.ro (M.-O.P.); adrianneacsu2006@yahoo.com (A.N.); 2 “Bucur” Maternity, Saint John Hospital, 012361 Bucharest, Romania; 3 Medicine Faculty, Lucian Blaga University, 550024 Sibiu, Romania; radu.chicea@gmail.com; 4 Obstetrics and Gynecology Dept, Iuliu Hatieganu Univerity of Medicine and Pharmacy, 400000 Cluj-Napoca, Romania; romeomicu@hotmail.com

**Keywords:** anorectal atresia, congenital anomaly, early prenatal diagnosis, ultrasound

## Abstract

Anorectal atresia (ARA) is a common congenital anomaly, but prenatal diagnosis is difficult, late, and unspecific. Utilizing a case of a 46 year old primipara with an egg donation In Vitro Fertilization (IVF) pregnancy, diagnosed at the first trimester scan with an anechoic isolated structure, which indicates anal atresia, we performed a systematic literature review in order to evaluate early prenatal ARA diagnosis. A total of 16 cases were reported as first trimester ARA suspicion, and only three had no associated anomalies. The most frequent ultrasound (US) sign was the presence of a cystic, anechoic pelvic structure of mainly tubular shape, or a plain abdominal cyst. In the majority of cases, structures were thin-walled and delimitated from the bladder. The presence of hyperechoic spots signifying enterolithiasis and peristaltic movements were helpful in order to establish the bowel origin of the lesion. Considering the high eventuality that the lesion is transitory, meaning later in pregnancy the fetus looks normal, early detection of such a sign should prompt further structural detailed evaluation, karyotyping, and appropriate pregnancy and postnatal counselling.

## 1. Introduction

An imperforate anus can be part of more complex congenital abnormal conditions that include anal atresia and anorectal atresia (ARA). The prevalence is high, ranging from 1/1500 to 1/5000 newborns [1]. The mechanism behind ARA is an impaired development of the urogenital septum that prevents the distal rectal pouch reaching the perineum, and also involves abnormal musculature development of the region (internal sphincter, external sphincter, and puborectalis muscle perianal muscular complex (PAMC)) [2].

ARA is associated in the literature with other malformations or chromosomal anomalies, at rates as high as 70% [3]. The most frequent associations are with trisomy 21, vertebral defects, anal atresia, cardiac defects, tracheo-esophageal fistula, renal anomalies, and limb abnormalities (VACTERL) syndrome, caudal regression syndrome, complex genitourinary malformations, and omphalocele-exstrophy-imperforate anus-spinal defects syndrome (OEIS) [1,3,4].

Prenatal diagnosis of ARA is difficult, and the signs are unspecific and indirect, including bowel dilatation and abnormal intestinal calcification [5]. There have been few cases of isolated ARA reported in the first trimester, and no systematic evaluation of the ultrasound (US) signs that should not be overlooked at this stage. The diagnosis is often made late in pregnancy or even after birth [6], although considering the morbidity, pathologic associations and impaired outcome, these babies could benefit from early recognition and proper counselling. More accurate examination and looking for the proper ultrasound signs (as described below) can improve the diagnosis rate in the first trimester). Among the most frequent complications of significant life-quality impact are fecal incontinence, bowel dysfunction, recurrent urinary infections, and sexual dysfunction [7].

The study was prompted by a case of a 46 year old primipara, with an egg donation In Vitro Fertilization (IVF), pregnancy presented for first trimester scan at 11 + 3 weeks. Other than an anechoic tubular structure in the fetal abdominal left quadrant, no other anomalies were noticed. The karyotype was performed and the result arr (1–22) x2, (XY) x1 was considered a normal variant. ARA was diagnosed and considered isolated since further detailed examination performed in the second and third trimester found no other anomalies. The baby was born at 36 weeks and low anorectal atresia with perineal fistula was diagnosed. Surgery was scheduled for three months of life. 

The aims of this systematic literature review were to assess the performance of the first trimester scan in ARA diagnosis and to indicate the most specific ultrasound signs of malformation, mainly when it is isolated, in the actual context of the first trimester ultrasound scan, thus expanding its capability of detailed analysis of the fetal anatomy. 

## 2. Materials and Methods

The analysis was performed according to the guidelines provided by Moher in 2009, indicated in “Preferred Reporting Items for Systematic Reviews and Meta-Analyses” as a PRISMA statement. No ethical committee approval was needed, given the type of study [8].

### Search and Information Sources

We searched the main databases, PubMed, Embase.com and Cochrane, electronically and with the following combination of key words and terms: “first trimester”, “early prenatal diagnosis”, “anal atresia”, “ano-rectal anomaly”, “ultrasound”, and “abdominal cystic anomalies”. All types of studies (retrospective, prospective, case control, systematic review) on ARA prenatal diagnosis were considered. The period included all papers published since 1990 and until 2020.

Inclusion criteria were: (1) papers describing ultrasound anomalies consistent with ARA suspicion/diagnosis, (2) cases reported in the first trimester of pregnancy (no further than 14 weeks), and (3) ARA diagnosis confirmed post-termination or postpartum. No language restriction was applied. 

The search and title evaluation were performed by two authors (LP and RMS) and assessed for the relevance of content. If the abstracts were relevant for the inclusion criteria, the full papers were extracted from the original publication. Differences of opinion were resolved by discussion with RC and RM. We excluded titles without full-text and images, and those where the diagnosis was performed after 15 weeks. After collecting the data, we excluded duplicated studies, and the remaining titles were analyzed based on the inclusion criteria. There was no need to contact the authors.

Statistical analysis was not applicable considering the qualitative evaluation and low number of reported cases. 

**Figure 1:**
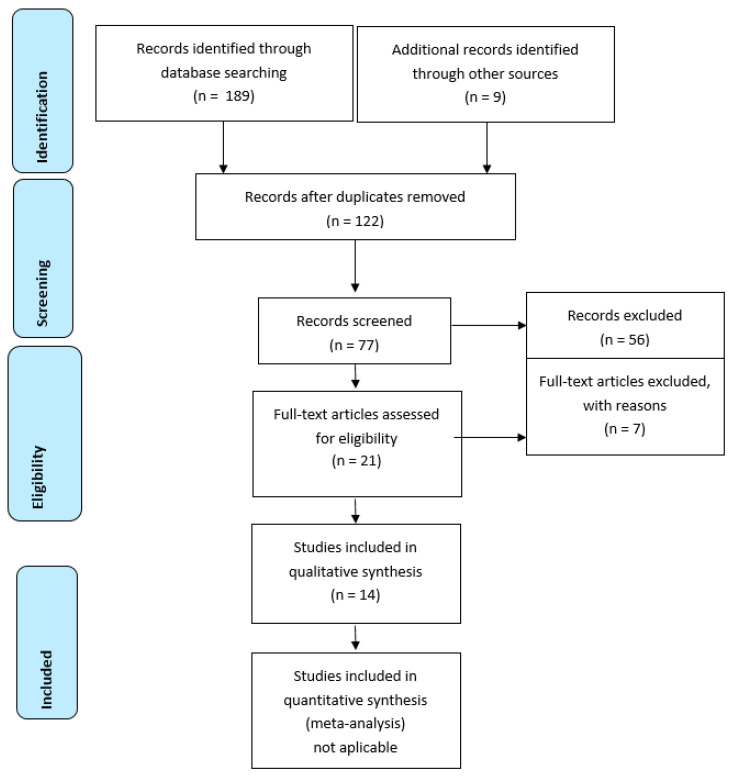
Flow diagram.

The database search provided 198 items, from which 76 were excluded as duplicates. After abstract assessment, 21 studies were retained and considered relevant for early prenatal diagnosis of ARA. Finally, seven papers were excluded for not reporting outcomes or not fulfilling the required criteria, and 16 cases of ARA diagnosed in the first trimester and confirmed postpartum or post pregnancy termination were extracted from 14 papers (two of them reporting two cases of early diagnosis) (Figure 1). To these we added our case, diagnosed at 11 + 6 weeks. The flow chart of the search is illustrated bellow. The main parameters that were analyzed are described in Table 1.

## 3. Results

The retained titles yielded a small number of prenatal ARA cases diagnosed in the first trimester; 16 cases, to which we added the case we diagnosed at 11 + 6 weeks. The maternal and paternal ages could not be statistically evaluated for correlation with the anomaly since not all papers reported them. Gestation age at diagnosis of ARA ranged between 11 + 2 weeks to 14 + 1 weeks. 

Fetal gender was not reported by all authors (three cases), but from the rest we noted a predominance of male fetuses (Male/Female (M/F) ratio 2.5/1). No title that reported signs of ARA referred to a twin pregnancy. Karyotyping was not performed in five cases, but did reveal a case of Trisomy 21 (T21), and in 11 cases was normal, including the case we reported, with arr (1–22) x2, (XY) x1 considered a normal variant.

Although, according to the inclusion criteria, the scans must be performed in the first trimester, in seven cases the nuchal translucency was not reported, and in the remaining cases, only one fetus had increased Nuchal Translucency (NT) reported as cystic hygroma. The other nine fetuses had NTs in the normal range. 

Ultrasound signs of ARA in the first trimester were described mainly as cystic structures: uni or multilocular; hypoechoic round, tubular, ovalar structures; hyperechoic structures in one case; located in the fetal abdomen/pelvis; delimitated from the urinary bladder. 

In four cases, the lesions disappeared later in the pregnancy, and in five cases, the second trimester scan was not performed. In the remaining seven cases, the lesions remained stable, progressed, or changed shape and aspect. Associated anomalies were reported in only 35% of cases, and consisted of ambiguous genitalia, spine anomalies, cloaca anomalies, kidney anomalies, and OEIS syndrome.

The pregnancy outcome was reported as live born in four cases, among which one was an emergency cesarean section (CS) for fetal distress at 29 weeks. One showed a true isolated ARA-associated perineal fistula, but with a very thin membrane covering the rectal pouch. The rest of the cases finished as termination of pregnancy (TOP) in the first trimester (two cases) or second trimester (nine cases). One was a spontaneous miscarriage.

## 4. Discussion

ARA is difficult to identify in the first trimester, when diagnosis relies on indirect signs. In assessing the first trimester detection on non-chromosomal anomalies, we found few reports on ARA, as Table 1 illustrates. The prenatal diagnosis rate of ARA detection is low (16%), even when considering diagnosis throughout the whole pregnancy period [6].

In an extensive study on the detection rate of different anomalies in 45,191 fetuses, Syngelaki reported no ano-rectal anomalies were detected [22]. This can be explained by the late first trimester development of the PAMC, so the absence of the anal sphincter could not be identified at the first trimester anomaly scan [2].

More recently, Rohrer evaluated the prenatal diagnosis of the ARA by ultrasound vs. magnetic resonance investigation (MRI) in 56 children over 10 years but did not report a diagnosis made below 20 weeks of gestation [23]. They concluded that, depending on the level of obstruction, the main sign of ARA is bowel dilatation and associated anomalies, and MRI is useful for correct identification of the fluid-filled pelvic organ (rectum, vagina, bladder, cloaca). Despite this important contribution to diagnosis, MRI is not indicated in the first trimester of pregnancy.

In 14 cases, the diagnosis was suspected due to finding cystic structures in the fetal abdomen and pelvis. These structures can take many forms but are mainly tubular shaped, and the aspect is determined by the associated anomalies (megacystis, rectovesical fistula, cloaca complex anomalies). A well-delineated, tubular shaped structure was indicative in our case. Additionally, the presence of hyperechoic structures inside the cysts can indicate bowel conditions, due to enterolithiasis [2]. In one case, the appearance was a hyperechoic pelvic structure, and in two cases of OEIS, the cystic structure was located at the level of the anterior abdominal wall and was diagnosed as omphalocel [4,13,15]. In our case, the hyperechoic structure (enterolithiasis) had odd movement that we interpreted as being produced by intestinal obstructed peristalsis.

Anechoic cystic structures are due to dilated bowels filled with liquid of uncertain origin. As a mechanism of obstructed bowel filling, it was proposed that fetal amniotic liquid is swallowed and, in some cases, contributes to the constitution of a rectovesical fistula. The accumulation of liquid can be explained since the rectum ends in a pouch, and the intestinal mucosa is not capable of absorption in early gestation [17].

Obviously, a cystic mass in the fetal abdomen prompts differential diagnosis with conditions other than ARA. Khalil et al., in a cohort retrospective study on 14 fetuses in which cystic structures were found at the first trimester anomaly scan, did not report any ARA but concluded that, considering the high probability that obstruction and gut malrotation produce bowel dilatation, this diagnosis should be included in differential diagnosis algorithms [24].

The literature reports that cystic images can be transitory in pregnancy, with spontaneous resolution in the second and third trimester mainly if the right colon is affected. The pathophysiology of right colon dilatation is not completely understood, and malrotation of the sigma or of the whole large bowel can be involved [9].

The presence of the fistulous trajectory reported in three cases can be explained by embryologic development. During cloaca and uroseptum formation, a crucial event is the recanalization of the “plug” that occludes the anorectal canal by apoptosis, a process that occurs in late embryologic development [25]. According to this theory, failure of recanalization or an improper process leading to abnormal orifices (fistula) can occur in early embryonic stages, while other ARAs with a normally placed anal orifice are a consequence of an injury and occur in a later stage. In our case, considering that the intestinal distension was observed early in the first trimester and disappeared later, and a perineal fistula was observed, we can suppose that the apoptotic process of anal recanalization occurred very early, and a second trimester evaluation only would have missed the anomaly. The decompression of the distended colon through a perineal or rectovesical fistula has also been proposed by other studies on cystic anomaly resolution in late gestation. The authors suggested that this transitory sign can be present in 37% of ARA cases, and it is useful to predict the condition from the first trimester [17].

The appearance of anomalies at the subsequent examination were reported not only as transitory findings, but also as the changing of echogenicity from anechoic to hyperechoic structures in two cases. There are two mechanisms that have been proposed for this hyperechogenic aspect of the obstructed bowel, some authors suggest that digestive enzymes accumulate in the colon after the first trimester and modify the composition of the intestinal contents. In normal fetuses, those enzymes are excreted in the amniotic fluid through a normal anal orifice [26]. Urinary content of oxalate and phosphate salts can also be responsible for the hyperechogenic aspect of dilated bowel contents in ARA associated with rectovesical fistula [27]. In our case, the anechoic initial cyst in the abdomen resolved in the second trimester, but the slightly echogenic rimmed rectum cannot be classified as a hyperechogenic structure, as reported by other papers. The possible explanation for this is that only a small amount of digestive enzymes accumulated after the fistula constitution, and this almost normal appearance of the lower fetal abdomen could have been misleading if no scan was performed in the first trimester.

Direct signs of ARA are not available in the first trimester due to the late development and difficult evaluation of the PAMC. The indirect signs of cystic pelvis structures cannot identify the level of obstruction but can allow an early diagnosis by contrary PAMC systematic evaluation, as Lee performed in a recent study. This could orientate the level of obstruction but had a sensitivity of only 74% [28]. On a large low risk pregnancies cohort (63,101 cases), Su evaluated the size and aspect of the anal canal and rectum and reported a high rate of prenatal isolated ARA diagnosis of 87.5%, but only after 20 weeks [29].

The etiology of ARA seems to be multifactorial, including some familial cases with autosomal dominant inheritance. Chromosomal abnormalities were only reported in one case of T21 in the case series (Table 1). Gene mutations involved in this condition include *SHH*, *EN2*, and *HLXB9*, which can be responsible for Pallister–Hall syndrome, Currarino syndrome, and Townes–Brocks syndrome, but are very rare [30]. In our case, the isolated finding led to a supposition that the anomaly was related to advanced paternal age (52 years) and the assisted reproductive procedure.

There is no consistent proof that, considering the minor gene polymorphism, the baby was not a carrier of a monogenic pathogenetic variant in our case (arr (1–22) x2, (XY) x1) that can be related to this anomaly. On the other side, the IVF procedures can be related to some genetic de novo anomalies and structural anomalies but so far there is no consensus in the literature on that issue [31].

The literature is scarce providing data for genetic causes of ARA. The studies include small samples and are mostly suspected to be gene approached, referring to *SHH*, *WNT*, and Fibroblast Growth Factor (FGF) signaling pathways as they are involved in multiple embryonic developmental processes. Multiple factors, such as genetic and environmental, are involved and genetic counseling is difficult since the risk of recurrence in unknown for non-syndromic ARA [32].

The cases reported in the literature found anomalies at the time of diagnosis in only six cases (ambiguous genitalia, single echogenic kidney, frontal bossing, skull demineralization, hemivertebra consistent with VACTREL, and caudal dysplasia). The postnatal or postmortem diagnosis indicated more syndrome cases (nine out of 12 cases), involving mainly the urogenital and cloacae-derived structures.

Considering that urogenital anomalies are a frequent association of ARA, Perlman evaluated 245 fetuses referred to a tertiary center for Congenital anomalies of the kidney and urinary tract (CAKUT) and found four cases with anal atresia, but only after 20 weeks. The detection rate was improved due to PAMC examination, and the authors highlighted that some of the cases could be overlooked because of the transitory signs of bowel dilatation after the first trimester [33].

The first anal atresia case identified in the first trimester by a US scan was reported by Girz as a part of OEIS, and the appearance was described as a large cystic structure on the anterior fetal abdomen (identified as omphalocel) [15]. In this case, the diagnosis was made after TOP triggered by spine anomalies. Other studies have reported other OEIS cases, but only three were picked up in the first trimester due to other associated anomalies [4,21].

Only five cases reported in the literature (including our case) were isolated anomalies, and this fact emphasizes the importance of first trimester diagnosis, which can possibly make the offer of genetic testing and close follow-up in pregnancy in order to correctly counsel and manage the situation [9,13,17,20].

An isolated anomaly, as in our case, and adequate fetal growth can allow pregnancy continuation until near term, and careful pregnancy evaluation is needed in order to assess the fetal wellbeing and to establish the appropriate birth modality.

There are some issues that further evaluation of ARA anomalies must address. First, there is a need to unify the terminology in order achieve consistent and reproducible reporting of cases (there are many synonyms, such as anal atresia, ano-rectal malformation, anal imperforation, etc.). Second, it is necessary to report maternal age, fetal sex, conception method, and other personal conditions such as obesity and diabetes, which could then be orientated towards the risk factors associated with ARA and prompt a detailed first trimester check.

## 5. Conclusions

A first trimester scan for detecting various pregnancy risks should be able to identify many fetal structural anomalies due to the high-resolution equipment and proximity of the fetal structures to the US transvaginal probe. Although isolated ARA is not a fatal disease, the physician should be aware that first trimester cystic or hypoechoic fetal pelvic structures trigger the diagnosis. Considering the transient character of those signs in the second trimester, when this condition can be overlooked, detailed ultrasound examination must be performed in order to exclude associated conditions and syndromes with an unfavorable outcome. Prenatal early diagnosis of isolated ARA does not change the prognosis of the pregnancy but offers the possibility of proper surgical counsel and immediate postpartum referral. The most sensitive issue in the couple’s counselling can be the context of advanced maternal age and Assisted Reproduction Techniques (ART) pregnancy, with a high likelihood of other pathological associations. In such a situation, precocity of the diagnosis makes further investigation possible and early identification of serious fetal anomalies informs TOP indication or confident pregnancy continuation.

## Figures and Tables

**Table 1:** Study characteristics.

Study, Author Type Year	Number of Reported Patients	Gestational Age at Diagnosis (Weeks)	Ultrasound Findings at Diagnosis	NT (mm)	Associated Anomalies	Fetal Gender	Genetic Assessment	Follow-Up Image Findings	Pregnancy Outcome	Neonatal/Post Termination Diagnosis
Bronshtein M CS 2017 [9]	2	13 + 4 14 + 0	transient, distended, and right-sided sigmoid colon transient, distended, and right-sided sigmoid colon	NR NR	Ambiguous genitalia, single echogenic kidney, frontal bossing, skull demineralization, hemivertebra No	NR NR	NP NP	NP Spontaneous resolution of cystic mass at 19 weeks.	TOP first trim Live born	Anal atresia confirmed, VACTERL Anal atresia confirmed
Carroll SG CR, 1996 [10]	1	12	Abdominal multiple cystic structure	NR	No	F	Normal 46 XX	Megacystis, hyper echogenic bowel, ascites, oligohydramnios.	TOP Sec trim	Female pseudo hermaphroditism with agenesis of the urethra, vagina, and rectum.
Chen M 2009 CR [11]	1	12	Multiple dilated bowel loops within the lower abdomen	1.9	NR—poor visibility due to maternal obesity	F	N 46 XX	NR—poor visibility due to maternal obesity	Spontaneous miscarriage sec trim	Atrogryposis multiples, CoA, univentriculr heart
Correia P CR, 2017 [12]	1	12	Hypoechoic tubular-shaped cyst was observed, on a retrovesical location, at the left lower abdomen.	NR	Abnormal male genitalia	M	N46XY	Dilated sigmoid showed intraluminal hyperechogenic foci suggestive for vesicorectal fistula	TOP sec trim	Anorectal agenesis, vesicorectal fistula, hypospadias
Dhombres F POS [13]	1	12 + 2	Hyperechogenic pelvic structure	1.9	No	NR	NP	Resolution of image at 17 weeks	Live born	Isolated imperforate anus
Gilbert A CR 2009 [14]	1	12 + 6	Cystic structure with a distal tapered appearance within the abdomen and pelvis of the fetus with an echogenic focus that did not exhibit echogenic shadowing	N	No	M	N	No anomaly at 24 weeks.	Emergency CS 29 weeks. –IUGR and fetal distress	Imperforate anus horseshoe kidney and low termination of the spinal cord at the third lumbar vertebral body.
Girz 2008 CR [15]	1	12 (?)	large cystic structure on the anterior fetal abdomen (identified as omphalocel).		Thoracic kyphoscoliosis	F	N46XX	Persistent anomaly 20 weeks consistent with OEIS	TOP 20 weeks	OEIS
Lam YH CR 2002 [16]	1	12	sausage shaped cystic mass (11 × 6 × 6 mm) in the right lower anterior abdominal cavity	1.4	No	M	N 46XY	No progression of the cystic mass, oligoanhidramnios	TOP Sec trim	anal atresia, malrotation of the gut, dilated sigmoid colon and rectum and a perimembranous ventricular septal defect
Liberty G SLR&CR 2018 [17]	1	13 + 1	cystic structure measuring 7 × 8 × 4 mm was identified in the right lower abdomen which tapered toward its distal part in the pelvis	1.7	No	M	N CGarray 46XY	16 weeks., cystic mass replaced by tubular shaped echogenic structure 21 weeks. absence of target sign (anal sphincter) prominent midline skin bridge in the fetal perineum	TOP sec trim	Absence of anal sphincter, high type ARA.
Mallman, M.R 2014 CR [4]	1	14 + 1	41 × 34 mm large cystic structure in the lower abdomen with	NR	Bladder extrophy, omphalocel OEIS	M	NP	NP	TOP 17 weeks	OEIS confirmed
Novikova I, CR 2011 [18]	2	11 + 2 11 + 3	dilated bowel within the lower abdominal cavity (10.0 mm × 2.0 mm) 8.4 mm anechogenic tubular structure in the lower abdomen (misdiagnosed as megacystis)	2.5 1.05	No No	F M	N 46 XX T21 47XY	16 weeks.–anhidramnios, no urinary bladder, no kidneys NP	TOP sec trim TOP early sec trim	Imperforate anus, rectal atresia, multiple anomalies compatible with Fraser syndrome Inconsistent due to tissue destruction during D&C (rectum dilatation?)
Santos J2013 Cr [19]	1	13	abdominal cystic formation	NR	caudal dysplasia with hypoplastic lower limbs	M	N 46XY	NP	TOP first trim	Multiple anomalies suggestive for VACTERL syndrome
Taipale P CR 2005 [20]	1	12	hypoechogenic cystic mass (14 × 7 × 8 mm) in the lower abdomen	1.1	No	M	NP	Similar findings as in first trim	Living birth	Anal atresia with fistula
Wax 2008 CR [21]	1	13	large multilocular cystic ventral wall mass measuring 4.7 3 4.0 3 3.5 cm	Cystic Hygroma	Thoracic hemyvertebrae	M	N46XY	16 weeks omphalocel splayed lumbosacral vertebrae and bilateral clubbed—OEIS	TOP 20 weeks	OEIS confirmed bifid phallus, symphyseal diastasis, imperforate anus, and no clear buttocks cleft

NT: nuchal translucency; F: feminine; M: masculine; N: normal; NP: not performed; NR: not reported; OEIS: omphalocele, exstrophy of the fetal bladder, imperforate anus, spinal anomalies; VACTERL syndrome: vertebral defects, anal atresia, cardiac defects, tracheoesophageal fistula, renal anomalies, and limb abnormalities; TOP: Termination of pregnancy; sec: second; CoA: Coarctation of Aorta; IUGR: Intrauterine Growth Restriction; ARA: Anorectal atresia; D&C: Dilatation and curretage.
